# Exact flow of particles using for state estimations in unmanned aerial systems` navigation

**DOI:** 10.1371/journal.pone.0231412

**Published:** 2020-04-15

**Authors:** Erol Duymaz, A. Ersan Oğuz, Hakan Temeltaş

**Affiliations:** Control and Automation Engineering Department, Istanbul Technical University (ITU), Maslak, Istanbul, Turkey; Universiti Sains Malaysia, MALAYSIA

## Abstract

The navigation is a substantial issue in the field of robotics. Simultaneous Localization and Mapping (SLAM) is a principle for many autonomous navigation applications, particularly in the Global Navigation Satellite System (GNSS) denied environments. Many SLAM methods made substantial contributions to improve its accuracy, cost, and efficiency. Still, it is a considerable challenge to manage robust SLAM, and there exist several attempts to find better estimation algorithms for it. In this research, we proposed a novel Bayesian filtering based Airborne SLAM structure for the first time in the literature. We also presented the mathematical background of the algorithm, and the SLAM model of an autonomous aerial vehicle. Simulation results emphasize that the new Airborne SLAM performance with the exact flow of particles using for recursive state estimations superior to other approaches emerged before, in terms of accuracy and speed of convergence. Nevertheless, its computational complexity may cause real-time application concerns, particularly in high-dimensional state spaces. However, in Airborne SLAM, it can be preferred in the measurement environments that use low uncertainty sensors because it gives more successful results by eliminating the problem of degeneration seen in the particle filter structure.

## Introduction

Autonomous systems have quite an importance for decision-makers and operational agents due to a large variety of its applications such as reconnaissance and surveillance in the military and civilian areas and their other extensive usage. The primary intended operation of operator-free systems is mainly autonomous navigation that provides flexible functionality, notably on a standalone service. It is crucial to determine the autonomous vehicle's precise position for better navigation in the operator-free services.

Global Navigation Satellite System (GNSS) is the preferred geolocation tool almost throughout the world. It is also mostly an adequate tool for an Unmanned Aerial Vehicle (UAV) position determination process. However, it can be hard to have successful results for this (localization) process in GNSS denied environments which may occur for several reasons. Thus, there have been many works in the literature about this problem recently. In GNSS denied environments (if the map knowledge does not exist either), the problem of concurrent position determination and production of map information around can be solved by Simultaneous Localization and Mapping (SLAM).

The autonomous platforms may operate in environments such as indoor, outdoor, underground, underwater, in changing conditions that include non-static, deformable/moving objects by using different sensors such as laser scanners, cameras, and many others. Many statistical or optimization-based SLAM methods recently made substantial contributions for improving its robustness in such conditions. However, it is still a considerable challenge to manage accurate, cost-efficient, robust SLAM. There exist several attempts to find better estimation algorithms for SLAM, and an essential part of its operations is the state estimations under uncertainty.

This study looks for a better alternative of recursive state estimations under uncertainty for the problem of Airborne Simultaneous Localization and Mapping (A-SLAM). Despite their success points, the prevalent systems (particularly Extended Kalman and Particle Filters) used in A-SLAM could produce a solution neither for drift problems that exhibit non-gaussian structure nor particle degeneracy problems that inhibit exact convergence. Since these problems crucial for autonomous navigation, we propose to use the 'Exact Flow of Particles' approach for A-SLAM state estimations and hope to solve them and find out better results.

The following parts will include related works, the exact flow of particles`theory and its A-SLAM application, simulations, results, discussion, and conclusion, respectively.

### Literature review

The SLAM concept was proposed by Smith-Cheesman [1], and conceptualized by researchers such as Dissanayake [2], Bailey [3]. Then Durrant-Whyte [4] has put forward the basis of the SLAM approach. In very first studies, Kalman Filter (KF) based approaches were (obviously) prevalent. With time-related ones emerged; the effect of observability on the Kalman filter-based SLAM structure investigated by Sukkarieh et al. [5], and stability and consistency problems were studied by Roumeliotis et al. [6]. Then other SLAM sub-problems were analyzed, and some solutions were proposed [7]. It was also possible to find other than territorial platforms’ applications of these SLAM researches [8] in which the most accurate positioning and mapping (on air) for autonomous navigation were critical [9].

SLAM researchers have used parametric filter approaches for estimation, such as Extended Kalman Filter (EKF) and Unscented Kalman Filter (UKF). They also used other derived non-parametric methods targeting better performance as well as Particle Filter (PF) [10]. In this context, Tong et al. were among them who have focused on image processing-based methods [11]. Yan et al. [12] proposed a particle filter-based SLAM construction on a robot platform. They also performed a real-time experiment to prove the effectiveness of their algorithm for the mobile robot in the dynamic laboratory environment. Meanwhile, some others have focused on the solution of the high processing burden of variables such as image size and platform dimension [13].

In recent years accelerated or parallelized filter structures have begun to be widely introduced [14]. While SLAM estimation methods differ according to the environment and vehicle type, the first SLAM studies were performed on less complex land vehicles. Later approaches that produce fewer errors due to the improved filter structure have also been applied to humanoid robots [15], and then marine/naval and aerial platforms [16,17]. Oguz and Temeltas [18] constructed an extended Kalman filter-based SLAM structure for UAVs. They presented the inconsistency problem in A-SLAM filtering for the first time with statistical and simulation results. They discussed the solution to the problem of inconsistency caused by the filter structure.

The observability, stability, and consistency of filters that used for estimations in uncertain environments have emerged as other active research topics for some time [19,20]. Julier and Uhlman [21] have observed that the estimated robot position error is reduced by repeated measurement of robot and landmarks`(pointers) positions.

It has been shown by Castellanos et al. [22] that the filter is inconsistent due to linearization errors in Kalman-based SLAM methods. Zhang et al. [23] investigated the convergence and consistency properties of an EKF-based SLAM structure, and observed that the filter jacobian should be full rank for a consistent estimation. Huang et al. [24] also optimized the Jacobian matrix used by the Lagrange polynomials method to reduce the distortion effect of unobserved subspaces on the filter to increase the consistency of the extended Kalman filter-based SLAM structure for territorial vehicles, and created SLAM structure with increased consistency by limiting observability.

The researchers utilized different types of (external-internal) sensors such as camera, laser, ultrasonic, RADAR, LIDAR to get (position) observation data in unmanned vehicles. Bender et al. [25] used camera and Inertial Navigation System (INS) data for the UAS`SLAM. Yang [26] designed the particle filter-based SLAM architecture for robots using ultrasonic sensors and also proposed a four-point object map verification technique (for mapping). It was resulting in increased accuracy of the algorithm and reduced mathematical processing burden. It is also possible to find works on sub-mapping and loop-closure problems in the literature [27].

Among real-time SLAM researches, Steckel et al. [28] performed an experimental study. They combined a biomimetic navigation model with a biomimetic sonar mounted on a mobile robot. They solved a simultaneous localization and mapping task, and proposed its applications in the field of biology as a model for sonar-based spatial orientation and map building. In this context, Guivant et al. [29] aimed for a real-time SLAM solution for an autonomous system, only using the filtering-based approach rather than graph optimization.

SLAM studies, some of which aimed at cooperative tasks called formation or swarm, have also been presented on the navigation of multiple vehicles and systems. The communications of these agents [30] are also problem areas in recent years [31].

Over the years, the researches of performance comparison of methods that have emerged before significantly increased as well [32]. Sualeh et al. [33] recently have projected into future potential SLAM studies as well as scanning past studies [34]. Huang [35] screened and analyzed the studies of SLAM (optimization) strategies from the outset, Yavuz et al. [36] compared EKF, UKF, and Fast SLAM performances. Zhang et al. [37] compared the Kalman filter and particle filter approaches in autonomous navigation in terms of technical principle and accuracy.

The particle flow filter approach was first proposed by Daum-Huang [38]. While Ding and Coates [39] used this approach in their research, Jilkov et al. [40] compared particle flow filter performances with parallel run particle filter [41]. The modified particle flow filter algorithms for different problems were also issued in time [42–45]. Although, particle flow method gave good results for usually sensor fusion, target tracking problems [38–45], adaptation and application of the exact flow of particles in autonomous navigation as filter structure of the Airborne SLAM problem has not been found in the literature yet, and this study will be the first by entitling it; Particle Flow Filter Based Simultaneous Localization and Mapping (PFF-A-SLAM).

## The exact flow of particles for non-linear filtering and particle flow filter based SLAM structure

Non-linear filtering, especially for high-dimensional systems`state estimation, is still a significant problem (particularly) because of the failure points in previous filter performances [38]. Of all-recursive state estimations, even in prevalent EKF and PF filter structures, a successful prediction and update process through the system and observation models aims to produce posterior (state values) close to real ones. However, such approaches may experience some drawbacks because of reasons such as the presence of non-gaussian noise, nonlinearity, or high dimensional systems`structure.

The (Exact) Flow of Particles for Non-linear Filtering has provided a novel approach by arousing interest in solving the curse of dimensionality of the particle filter for state estimation in non-linear systems [39]. Daum and Huang [38] introduced it earlier as Particle Flow Filter (PFF) or Daum-Huang Filter (DHF) as an alternative filtering way such that a homotopy which defines a particle flow introduced between the logarithms of the unnormalized prior and posterior densities as the solution to a partial differential equation (at each time step of filter operations). The particle flow causes particles to migrate to regions where the posterior is high in value. By this approach, after estimation/prediction, a more rapid and exact update step emerges in the processes. There have been several PFF method versions proposed by Daum. However, reliance on parallel EKF or UKF exists for some, such as the exact flow of particles`filter [38].

### Particle flow filter evolution

In order to better understand the contribution of the particle flow filter, its historical development process should be examined. The first common filter structure-EKF is a parametric predictor and smoother. Whereas assuming (system and observation) uncertainties as gaussian, it estimates the state, observation, and covariance using a linearized system model (state-transition). Then it updates them, and this cycle continues in a recursive manner. EKF, satisfied with the convergence and (process) speed/workload optimization (also features of robustness, sensitivity, commonness) in many problems applied overtime after its appearance. However, it has become an approach for which researchers seek a continual alternative for long because of the performance losses (such as consistency drawbacks, observability problems) experienced in high dimensional/complex systems because of applying linearization to nonlinear systems. More importantly, of the uncertainty convergence success that it cannot show in systems with non-gaussian uncertainty.

Then UKF, which was similar in some way to EKF structure but differently that did not use the linearization approach, was developed. The UKF has operated by defining the sigma points around the state. It estimates the state (set), observation, and covariance in accordance with their weights. Then again, using the weighted sigma points, it updates the evaluation of the parameters such as the innovation, correlation matrix, Kalman gain, and the completed cycle continues recursively. The UKF structure improves estimation convergence, consistency, and observability. Nonetheless, it has not been able to produce a solution (especially) to the convergence problem in systems where uncertainty is not gaussian.

After that, the PF introduced as the solution to this problem. It employs any probability distribution with m values determined around the initial state and each sampled value in this distribution functions as a particle. The prediction performed through the state transition function is repeated for all the particles. According to the observation results, data association (the calculation of which particle corresponds to how much weight) is conducted. Then all these weights are normalized. The weights of particles, whose predictions much closer to the observation, will be higher (importance sampling). Later, a new particle distribution can be obtained by using the cumulative sum of the samples (sample histogram) in the current distribution (i.e., roulette chart method).

Meanwhile, other different methods could be used in the re-sampling process. In this distribution, the ones with the highest weight will occupy more than the previous ones. In this case, while some particles disappear, some particles continue as stronger to the iterations. However, after a while, these survivor particles (even if different observation results come back) do not change, and it causes degeneration. Although PF has created excitement with its success in non-gaussian uncertainties, it has not been able to reach the final solution in the estimation problem. More searches have continued, mainly because of the particle degeneracy, which has been affecting the success of convergence.

At this point, the PFF emerged by claiming the idea of (being) an optimal, stable, fast, and accurate estimator. It used a similar particle approach in the PF structure. However, it does not rely on using weighting and re-sampling steps of the produced particles (after their estimation) depending on the observation. It provides the fastest convergence to the particles with the highest probability of being terminated in the next step. To make it employs a defined logarithmic-homotopy-function (based on the prior probability and observation). The details of its operation are explained in the next section. The PFF eliminated the particle degeneration seen in the PF structure while performing this fast and accurate convergence by giving more successful results in environments using more deterministic/low uncertainty or high-quality sensors.

### Particle flow filter theory

The Particle Flow Filter uses a set of homotopy functions that define transitions between prior and posterior probability density distributions of the flows of particles. It uses the solution of a set of differential equations (Itostochastic Partial Differential Equation-Ito PDE) defined between the step (size) parameter and the state.

The PFF method refers to the solution of this equation in a Fokker-Plank approximation. It provides particles’ flows (migrations) to high the posterior probability zone in the quantities determined by the solution of the Fokker Plank equations [38].

In the PF structure, there were recursive processes of the estimation step (estimation of each particle) using the motion/prediction pattern of n particles sampled from the (normal) distribution around the state itself, starting from the prior probability space. The processes of predictions in which the best (closest estimating) particles continue to perform iterations/operations more strongly, based on (observation) estimation error. The PFF here makes an improvement that allows the particles to converge faster to the strongest particles by means of a defined homotopy function (detailed below).

**Fig 1:**
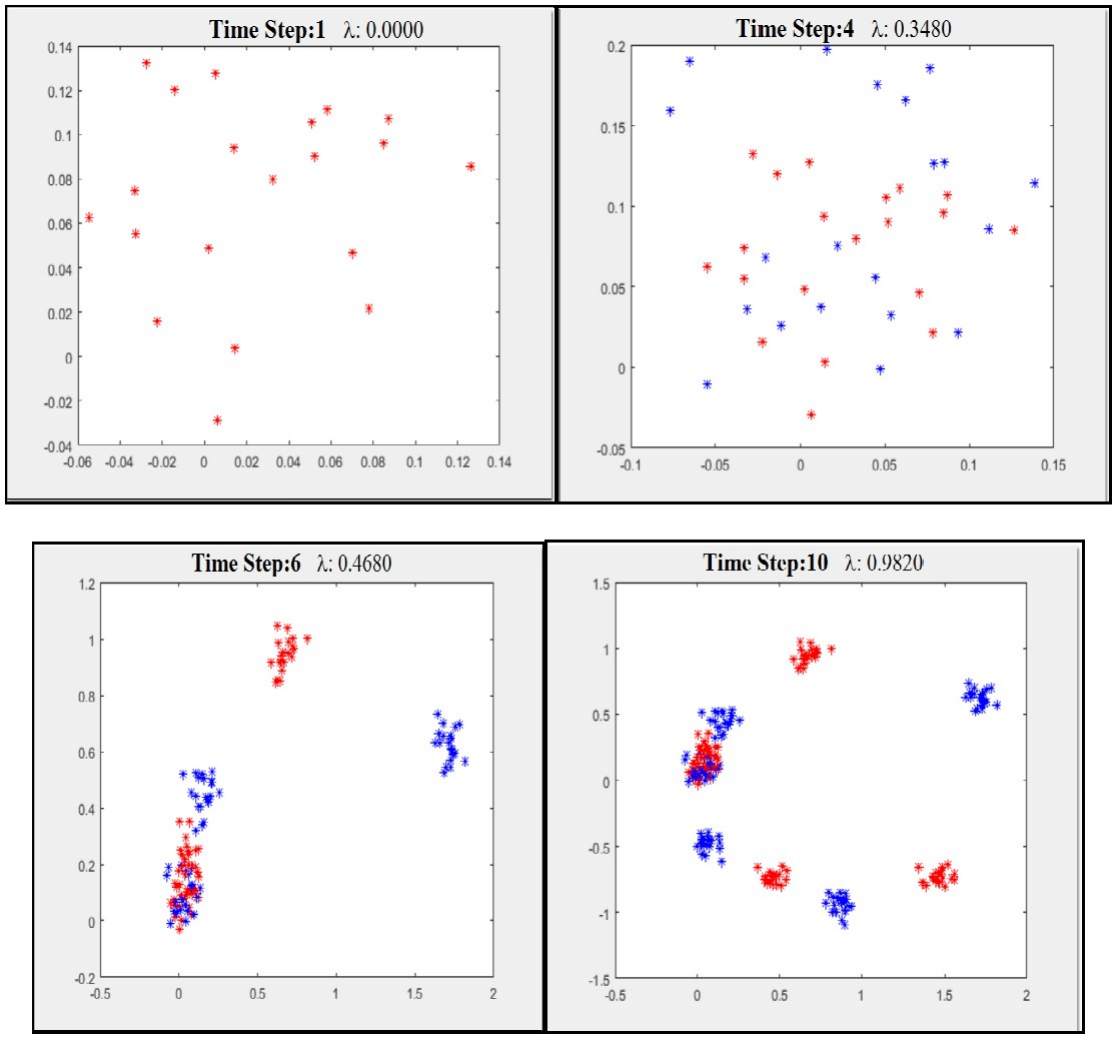
Exact flow of particles for nonlinear filtering—The algorithm steps outlook.

The particle flow structure at the beginning and end of an estimation step period is given as an example in Fig 1. Here, some details about a small number of particles and the function of the lambda step parameter (choosing big steps between 0–1 as to see particle flows/migration better) in the transition from prior probability to posterior probability (particle flows/migration) are given through pictures.

For PFF mathematics, Bayes Rule, as remembered, is used to define the marginal posterior probability density that is not normalized [38];

$$p(x_{t}\left|y_{1:t})=p(y_{t}\right|x_{t})p(x_{t}|y_{1:t-1})$$ (1)

where;

$$h(x_{t})=p(y_{t}|x_{t})$$ (2)

$$g(x_{t})=p(x_{t}|y_{1:t-1})$$ (3)

(respectively) h and g are the likelihood (observation) and the prior [38], and a logarithmic homotopy function to be defined for the relation between the final (posterior) and prior probability densities as;

$$\text{log}\phi (x_{t},\lambda )=\text{log}\;g(x_{t})+\lambda \;\text{log}\;h(x_{t})$$ (4)

If λ is defined between 0 and 1 (real value and continuous), it will be a step parameter that determines the amount of particle flow/migration. Here, the logarithm of the final posterior probability density is the logarithm of prior density log g (x_t_) if (λ = 0). It is the log (sum) of the prior and likelihood values when (λ = 1). The evidence marginality value is here included in calculations as a normalization factor.

If we remove logarithms from both sides of the equation, it is seen that the product of the prior and likelihood is equal to the posterior value as of the Bayes rule. In this context, it is also clear that the filter and step-size parameter of it work as a factor of weighting or reliance between prior and likelihood participation for the next state. As from this point (for the step size ranging from 0 to 1), the posterior probability only consists of the prior probability weight by not taking evidence into account for (λ = 0). The posterior (final) probability is formed by the prior probability and observation together with equal weights for (λ = 1).

dx=ψ(x,λ)dλ+dΩ (5)

$$\left(\frac{dx}{d\lambda })=\psi (x,\lambda \right)$$ (6)

It is assumed that the (generic) ito-stochastic differential equation (in Eq 5) will represent the flow change between the step size and the state in the homotopy function. That is a differential equation with a statistical term in its structure and used in physical flow problems whose solutions are also a stochastic process.

According to this expression and the homotopy given by Eq (4), the flow of particles is in the direction of the directional gradient of the logarithmic homotopy function. The particles flow at the rate proportional to the logarithmic observation (h) and stop at the point where the gradient equals zero.

Here, according to the equation of zero diffusion, which accepts that there is no noise term in Eq (5), the notation becomes the form in Eq (6). It is assumed here that ito-stochastic equation which determines the particle flows at this point can be found by using the ordinary differential equation (ODE) of Fokker-Planck (the time-varying expression of the pdf representing the particle velocity under drift and random effects) given by Eq (7) [38];

$$\frac{d\phi }{d\lambda }=-div(\psi \phi )+0.5div(\text{P}(x)\frac{\partial \phi }{\partial x})$$ (7)

$$(\frac{\partial \phi }{\partial x}\frac{d\phi }{d\lambda })+\frac{d\phi }{d\lambda }=0$$ (8)

Here, if the equation is modified according to zero diffusion (P process covariance is 0), then the Eq (8) is formed (Exact Particle Flow), and its solution with Eq (4) specifies the probability density flow as;

$$\frac{\partial \phi }{\partial x}\psi (x,\lambda )+\text{log}(h)=-div(\frac{d\psi }{dx})$$ (9)

When the partial differential equation (PDE) given by Eq (9) is solved, the values of A and b affecting the amount of flow in the particles are reached as;

$$A=(-0.5)PH^{T}{\left(\lambda HPH^{T}+R\right)}^{-1}H$$ (10)

$$b=(I+2\lambda A)[(I+\lambda A)PH^{T}R^{-1}y+A\bar{x}]$$ (11)

and here the flow quantities in each iteration of the particles are formed as follows [38];

$$\left(\frac{dx}{d\lambda })=A(\lambda )x+b(\lambda \right)$$ (12)

### Particle flow filter based airborne SLAM (PFF-A-SLAM)

In this section, we describe our proposed A-SLAM algorithm based on the exact flow of particles in the filter structure. The approach can be stated as the PFF-A-SLAM algorithm, which is used for aerial systems, and has prediction and update steps as well for autonomous navigation procedures. It is formulated using a Particle Flow Filter (PFF) in which map markers’ locations and the vehicle’s position, velocity. The Euler angles here are estimated using relative observations between the vehicle and each landmark/marker.

#### Non-linear prediction and observation model

Air vehicle state vector and map vector form the state vector used in the non-linear system model. Air vehicle state vector is composed of the (x,y,z) positions *P*^*n*^(*k*), (Vx,Vy,Vz) velocities *V*^*n*^(*k*) and the Euler (roll, pitch, and yaw) angles Ψ^*n*^(*k*).

The map vector is made up of the position of each landmark (x_L_, y_L_, z_L_). The general phrase for the length of this vector is mxn (m: number of landmarks, n: position dimensions of each landmark).

$$x(k)=\left[\begin{array}{c} x_{UAV}\\ x_{MAP} \end{array}\right]$$ (13)

The system (state-space) model general expression used in filter and observation model of air vehicle at step k is stated explicitly as;

x(k+1)=f(x(k),u(k),v(k)) (14)

z(k+1)=h(x(k),ω(k)) (15)

where u(k) is control input of the system, v(k) and w(k) are zero mean white noises, their covariances are *Q* = [*v*(*k*).*v*(*k*)^*T*^] and *R* = [*ω*(*k*). *ω*(*k*)^*T*^].

In the state-space model, the state vector at the step of k+1 depends on state vector and input data of step k. The landmark positions here are stated through the range, bearing, and elevation, as in the observation model.

The transformations that value from the sensor frame to the navigation frame are performed by the transformation matrix given in Simulation Model Background in Section 4. The state-space model in explicit equation form can be written as;

$$F_{UAV}=\left[\begin{array}{c} P^{n}(k)\\ V^{n}(k)\\ \Psi^{n}(k) \end{array}\right]=\left[\begin{array}{c} P^{n}(k-1)+\Delta t*V^{n}(k-1)\\ V^{n}(k-1)+\Delta t*T_{b}^{n}(k-1)*f^{b}(k)]\\ \Psi^{n}(k-1)+\Delta t*E_{b}^{n}(k-1)*w^{b}(k) \end{array}\right]+\left[\begin{array}{c} w_{P^{n}}\\ w_{V^{n}}\\ w_{\Psi^{n}} \end{array}\right]$$ (16)

where $f_{x,y,z}^{b}$ and $w_{\varphi ,\theta ,\psi }^{b}$ represents body frame directional velocities and angle variance in related axis accordingly, and $T_{b}^{n}$ and $E_{b}^{n}$ body to navigation frame transformation matrix.

The system Jacobian matrix which is composed of motion model`s partial derivatives of vehicle positions, rates and the Euler angles as below explicitly/closed formula;

$$\nabla f_{uav}(k)=\left[\begin{array}{ccc} \frac{\partial P^{n}(k)}{\partial P^{n}(k-1)} & \frac{\partial P^{n}(k)}{\partial V^{n}(k-1)} & \frac{\partial P^{n}(k)}{\partial \Psi^{n}(k-1)}\\ \frac{\partial V^{n}(k)}{\partial P^{n}(k-1)} & \frac{\partial V^{n}(k)}{\partial V^{n}(k-1)} & \frac{\partial V^{n}(k)}{\partial \Psi^{n}(k-1)}\\ \frac{\partial \Psi^{n}(k)}{\partial P^{n}(k-1)} & \frac{\partial \Psi^{n}(k)}{\partial V^{n}(k-1)} & \frac{\partial \Psi^{n}(k)}{\partial \Psi^{n}(k-1)} \end{array}\right]$$ (17)

or in the structural formula;

$$\nabla f_{uav}=\left[\begin{array}{ccccccccc} 1 & 0 & 0 & \Delta t & 0 & 0 & 0 & 0 & 0\\ 0 & 1 & 0 & 0 & \Delta t & 0 & 0 & 0 & 0\\ 0 & 0 & 1 & 0 & 0 & \Delta t & 0 & 0 & 0\\ 0 & 0 & 0 & 1 & 0 & 0 & \Delta t\frac{\partial (T_{b}^{n}(k-1)f_{x}^{b}(k))}{\partial \varphi } & \Delta t\frac{\partial (T_{b}^{n}(k-1)f_{x}^{b}(k))}{\partial \theta } & \Delta t\frac{\partial (T_{b}^{n}(k-1)f_{x}^{b}(k))}{\partial \psi }\\ 0 & 0 & 0 & 0 & 1 & 0 & \Delta t\frac{\partial (T_{b}^{n}(k-1)f_{y}^{b}(k))}{\partial \varphi } & \Delta t\frac{\partial (T_{b}^{n}(k-1)f_{y}^{b}(k))}{\partial \theta } & \Delta t\frac{\partial (T_{b}^{n}(k-1)f_{y}^{b}(k))}{\partial \psi }\\ 0 & 0 & 0 & 0 & 0 & 1 & \Delta t\frac{\partial (T_{b}^{n}(k-1)f_{z}^{b}(k))}{\partial \varphi } & \Delta t\frac{\partial (T_{b}^{n}(k-1)f_{z}^{b}(k))}{\partial \theta } & \Delta t\frac{\partial (T_{b}^{n}(k-1)f_{z}^{b}(k))}{\partial \psi }\\ 0 & 0 & 0 & 0 & 0 & 0 & 1+\Delta t\frac{\partial (E_{b}^{n}(k-1)w_{\varphi }^{b}(k))}{\partial \varphi } & \Delta t\frac{\partial (E_{b}^{n}(k-1)w_{\varphi }^{b}(k))}{\partial \theta } & \Delta t\frac{\partial (E_{b}^{n}(k-1)w_{\varphi }^{b}(k))}{\partial \psi }\\ 0 & 0 & 0 & 0 & 0 & 0 & \Delta t\frac{\partial (E_{b}^{n}(k-1)w_{\theta }^{b}(k))}{\partial \varphi } & 1+\Delta t\frac{\partial (E_{b}^{n}(k-1)w_{\theta }^{b}(k))}{\partial \theta } & \Delta t\frac{\partial (E_{b}^{n}(k-1)w_{\theta }^{b}(k))}{\partial \psi }\\ 0 & 0 & 0 & 0 & 0 & 0 & \Delta t\frac{\partial (E_{b}^{n}(k-1)w_{\psi }^{b}(k))}{\partial \varphi } & \Delta t\frac{\partial (E_{b}^{n}(k-1)w_{\psi }^{b}(k))}{\partial \theta } & 1+\Delta t\frac{\partial (E_{b}^{n}(k-1)w_{\psi }^{b}(k))}{\partial \psi } \end{array}\right]$$ (18)

The observation Jacobian matrix which is composed of measurement model`s partial derivatives of landmark/feature and vehicle positions, and the Euler angles as below (again explicitly);

$$\nabla h=H=\left[\begin{array}{ccc} \frac{\partial \rho (k)}{\partial P^{n}(k)} & \frac{\partial \rho (k)}{\partial V^{n}(k)} & \frac{\partial \rho (k)}{\partial \Psi^{n}(k)}\\ \frac{\partial \phi (k)}{\partial P^{n}(k)} & \frac{\partial \phi (k)}{\partial V^{n}(k)} & \frac{\partial \phi (k)}{\partial \Psi^{n}(k)}\\ \frac{\partial \vartheta (k)}{\partial P^{n}(k)} & \frac{\partial \vartheta (k)}{\partial V^{n}(k)} & \frac{\partial \vartheta (k)}{\partial \Psi^{n}(k)} \end{array}\right]$$ (19)

#### Predictions

**Fig 2:**
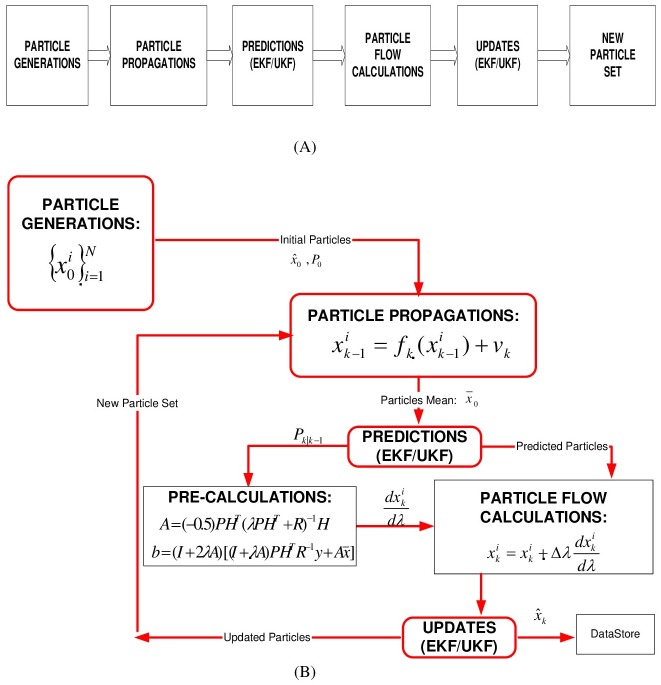
Particle flow filter based A-SLAM algorithm. (A) General block diagram. (B) Flow chart diagram.

An unmanned aerial vehicle`system and observation models are essential besides regular PFF operations for the algorithm given in Fig 2. The states in this work are determined as parameters of vehicle positions, directional velocity (rates), and Euler angles in addition to map vector (landmark positions) in reference to different axes. The predictions of (generated) particles performed based on the system model and updated according to PFF flow migration algorithms.

*Particle generations/initializations*. The initial particles are generated randomly. The particle set here is characterized by first and second moments, as well as the number of particles. For the next cycles of the algorithm, the particle generations are determined by previous particle update steps and EKF/UKF covariances.

*Particle propagations and predictions*. Particle propagations are conducted according to the system model given with Eq (16). Because of the parallel run EKF, we need for covariance predictions, Eq (20) below is used;

$$P(k+1|k)=\nabla F_{x}.P(k|k).\nabla F_{x}{}^{T}+\nabla F_{u}.Q.\nabla F_{u}{}^{T}$$ (20)

where ∇*F*_*x*_, ∇*F*_*u*_ are jacobians, *P* and *Q* are (measurement) covariance estimation and process noise covariance, respectively.

#### Updates

The update, which is performed by PFF migrations, is different than Kalman gain-innovation calculations in EKF and importance sampling steps in Particle Filter. For the PFF update process, migration calculations are needed first.

*Particle migrations*. Particle migrations are conducted according to PFF Eqs (10)–(12), and here for “x” state and “λ” step size, the parameter (dx/dλ) represents particles’ flows.

*Particle updates and re-generations*. The particles are updated after migration. Depending on the step size “λ” given above, the change of the x state variable (carrying the position information and migrating in each cycle) substantially determines the new state for the SLAM problem (dx/dλ-given above). While the variables A and b are the state variable and the other variable, P and R are covariances, and the H is the observation matrix. Here, (dx/dλ) is the parameter that determines the amount of flow for the particles, and x_k_ is the new state after particles flow.

$$x^{i}k=x^{i}k+\Delta \lambda \frac{dx^{i}k}{d\lambda }$$ (21)

Once a landmark has been initiated into the state vector, subsequent observations of this landmark are used to update the entire state vector consisting of the vehicle position, velocities and landmark positions (of this landmark and other landmarks) in the environment. Then the state estimation is updated using Eq (21).

The particle re-generations are needed for better particle convergence. Both for particle re-generations (based on P, H, R) and parallel-run EKF recursions, the EKF-updates are performed depending upon Eqs (22), (23) and (24);

$$P(k+1|k+1)=P(k+1|k)-W.S(k+1).W^{T}$$ (22)

$$S(k+1)=\nabla H_{x}P(k+1|k)\nabla H_{x}{}^{T}+R$$ (23)

$$W=P(k+1|k).\nabla H_{x}^{T}.S^{-1}(k+1)$$ (24)

where ∇*H*_*x*_, *W*, *S* are observation jacobian, Kalman gain, and innovation covariance, respectively.

Briefly, Fig 2 demonstrates PFF based aerial vehicle SLAM general block (for one cycle) and flow chart diagram structure. This algorithm is proposed here first time in the literature as Particle Flow Filter based Airborne Simultaneous Localization and Mapping (PFF-A-SLAM).

#### Other discussions

Although EKF is here preferred as a general approach to (noise) covariance information, UKF could have been used instead. Although EKF is here preferred as a general approach to (noise) covariance information, UKF could have been used instead. It is also possible to perform the (process and observation) covariance and matrix (P, H, R) calculations (differently) using various differential equation solutions without EKF/UKF as in incompressible flow (in Eq 7) or any other (such as irrotational, geodesic, stabilized, or small curvative flow) particle flow filter approaches [38]. In each of these, since different assumptions are made, they may differ in their accountability and performance [38]. As expected, these various particle flow calculations determine the PFF results, which will be discussed in the following section.

Here we perform PFF estimations`comparison with EKF and PF for SLAM problem. Thus, the Exact Particle Flow, one of several PFF versions that perform the iterations by parallel EKF/UKF run execution chosen here for the sake of simplicity and practicalness.

**Fig 3:**
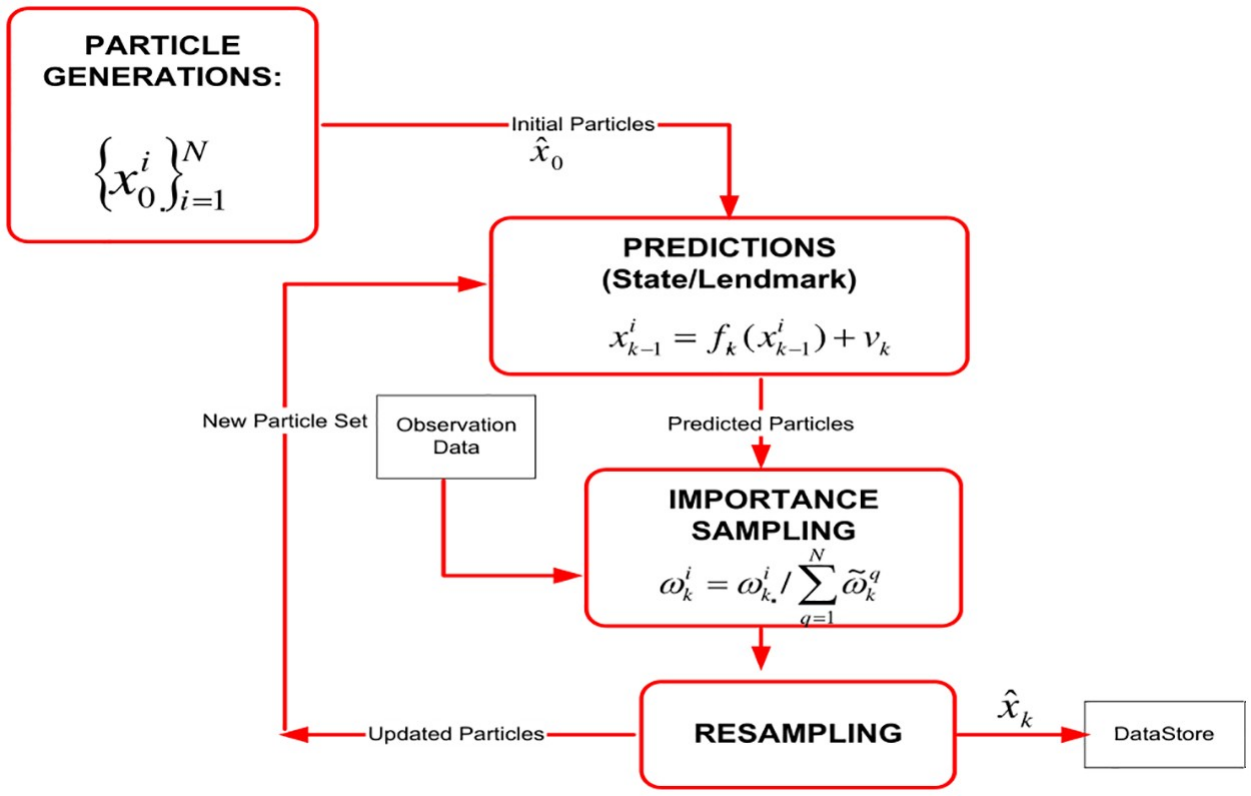
Particle filter based A-SLAM algorithm.

All the filter algorithms (PFF, PF, EKF) used in simulations are in-house manufactures developed by us according to the algorithms given in Fig 2 (for PFF), Fig 3 (for PF) and by the Eqs given in (20), (22), (23), (24), (25) and (26).

υ(k+1)=z(k+1)−z(k+1|k) (25)

$$x(k+1|k+1)=\hat{x}(k+1|k)+W.\upsilon (k+1)$$ (26)

In EKF, except the state and measurement predictions performed according to system and observation models Eqs in (14) and (15), and the covariance predictions are made by Eq (20). These all consist of EKF predictions and EKF updates are conducted by Eqs (22), (23), (24), (25) and (26).

## Simulations and results

### Simulation model background

The general system equation is expressed as the kinematic equation of the air vehicle. Its matrix form includes mathematics of motion without forces that affect the motion or relation between control parameters and the behavior of a system in space.

The transformation of the body frame motions of an air vehicle to the navigation frame plays a vital role since the global mapping of the environment requires mapping of air vehicle equations of motion to the global frame. Euler angle transformations are also to be used for this purpose.

**Fig 4:**
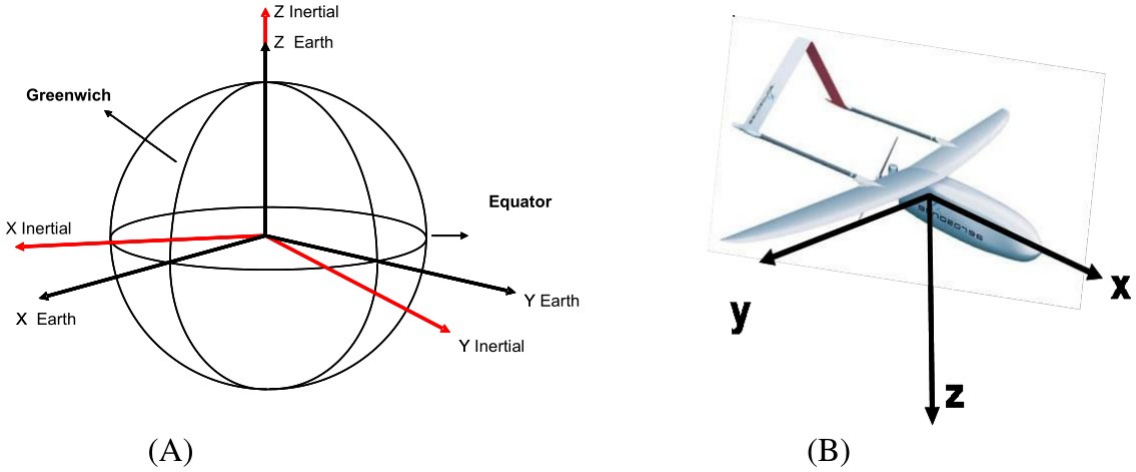
Coordinate systems. (A) Earth-centered fixed plane. (B) UAV body frame [18].

The air vehicle body frame (Fig 4) accelerations of the directional navigation and angular rates are transferred to the navigation frame. The position of the air vehicle is calculated in the navigation frame. The matrix expression obtained by the transfer of directional acceleration to navigation frame of air vehicles is;

$$\left[\begin{array}{c} \ddot{x}\\ \ddot{y}\\ \ddot{z} \end{array}\right]=\left[\begin{array}{ccc} \;\text{cos}\;\theta \;\text{cos}\;\Psi & \;\text{cos}\;\Psi \;\text{sin}\;\theta \;\text{sin}\;\phi -\;\text{cos}\;\phi \;\text{sin}\;\Psi & \;\text{cos}\;\Psi \;\text{sin}\;\theta \;\text{cos}\;\phi +\;\text{sin}\;\phi \;\text{sin}\;\Psi \\ \;\text{cos}\;\theta \;\text{sin}\;\Psi & \;\text{cos}\;\phi \;\text{cos}\;\Psi +\;\text{sin}\;\theta \;\text{sin}\;\phi \;\text{sin}\;\Psi & -\;\text{sin}\;\phi \;\text{cos}\;\Psi +\;\text{sin}\;\theta \;\text{cos}\;\phi \;\text{sin}\;\Psi \\ -\;\text{sin}\;\theta & \;\text{cos}\;\theta \;\text{sin}\;\phi & \;\text{cos}\;\theta \;\text{cos}\;\phi \end{array}\right].\left[\begin{array}{c} u\\ v\\ w \end{array}\right]$$ (27)

The transformation matrix of angular rates from the body frame to navigating frame is;

$$\left[\begin{array}{c} \dot{\phi }\\ \dot{\theta }\\ \dot{\Psi } \end{array}\right]=\left[\begin{array}{ccc} 1 & \text{sin}\phi \text{tan}\theta & \text{cos}\phi \text{tan}\theta \\ 0 & \text{cos}\phi & -\text{sin}\phi \\ 0 & \text{sin}\phi \text{sec}\theta & \text{cos}\phi \text{sec}\theta \end{array}\right].\left[\begin{array}{c} p\\ q\\ r \end{array}\right]$$ (28)

The vectors referred to in the kinematic model of air vehicle are given here as [u,v,w], which is directional acceleration. The expression [p,q,r] is angular rates in the body frame.

### Modeling and simulation environment of an aerial autonomous system

In this study, the estimation performance of the particle flow filter under uncertainty in the A-SLAM structure is examined. Thus, the approximate prediction of convergence in the most straightforward scenarios are evaluated with a vehicle and sensor model of the system based on a 6-degree of freedom for air vehicle structure.

**Fig 5:**
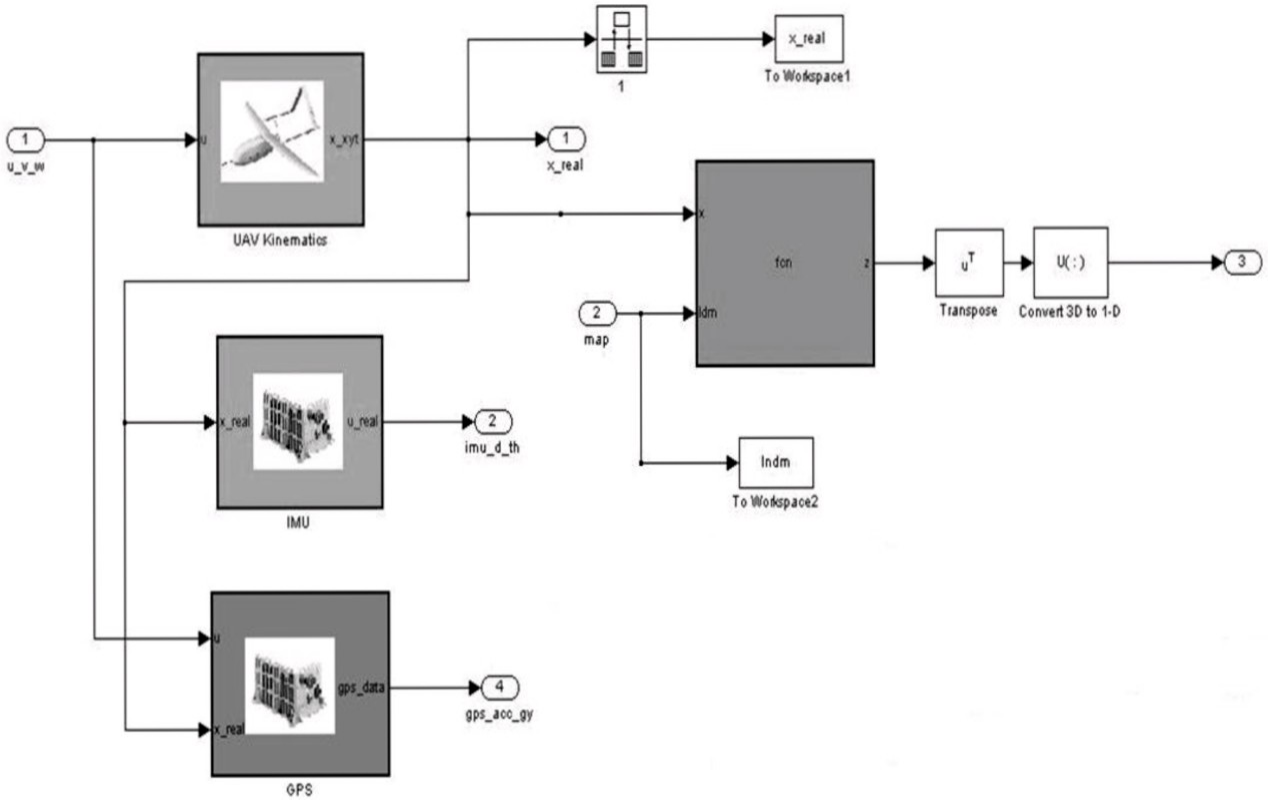
UAV simulation sub-structures.

**Fig 6:**
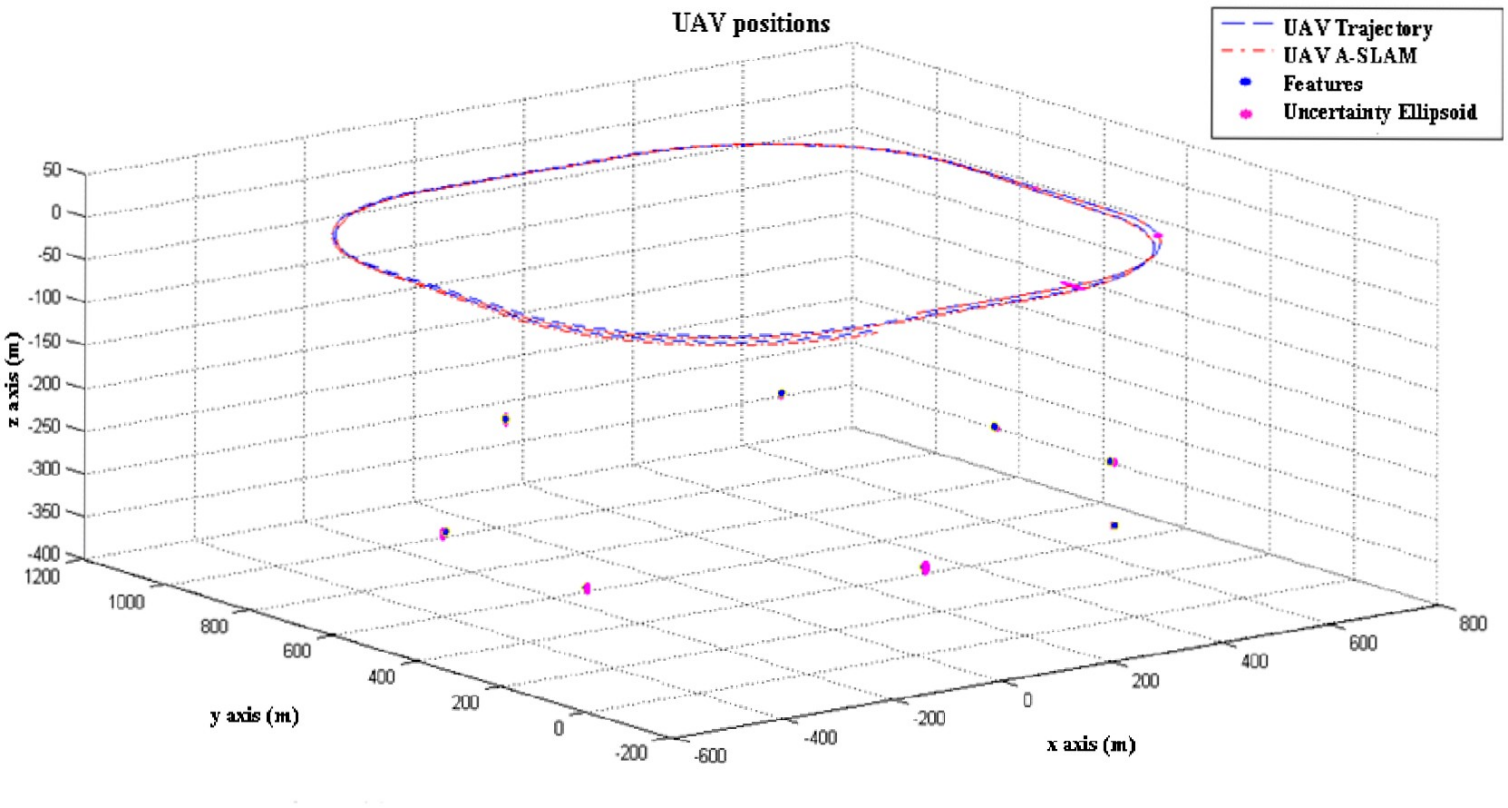
Simulation flight pattern.

In the algorithms developed in the simulation environment, it is assumed that a UAV (autonomous vehicle-simulation model in Fig 5) starts to move from an unknown point to an environment without a prior topological information or location information of the reference objects (landmarks) belonging to the environment. In the designed approaches, it is supposed to return to the starting point; the vehicle detects objects around it while navigating and decides where to go with the help of control inputs. In the integrated simulated scenario, the vehicle traveling at a constant airspeed at a specific/fixed altitude, as shown in Fig 6, making a circulatory flight on a smooth route.

In this context, it is assumed that the vehicle has not encountered any obstacles during flight. It has seen a certain number of landmarks at sufficient distance from each other throughout the path and that it has performed a loop closure by accurately identifying it.

Here, specific problems such as processing of the data coming from the sensors or ones about landmark observations are excluded from the evaluation. It is also assumed that data association, meaning that which observation value is known belonging to which landmark or that correspondings for all observations, are known.

## Results and comparisons

Although it is only summarized here, in order to determine the vehicle location and environment map with landmark object detection with the sensor information, various operations conducted. In several different simulation environments, including the different number of particles, flow step size, motion and measurement noises/uncertainties, vehicle and sensor number/position/types, navigation path and duration, and many more other various variables and scenarios to quantify performance employing different measures.

The numerical values obtained here are given for the same scenario above, such as the vehicle-sensor model given in Section 3, s_1_ = (0.01); meaning 1% motion/system; s_2_ = (0.1); meaning 10% sensor/measurement uncertainties (noise), and 500 number of particles. The same calculation hardware (which is Sony Vaio NW Series Notebook, has a 2.2GHz Intel Due Core i2 processor, 4 GB of 800MHz DDR2 memory, and an Intel GMA 4500 MHD 1750 MB graphics card) for a full loop closure and data association conditions of 1000 m circulatory flight pattern are provided.

**Fig 7:**
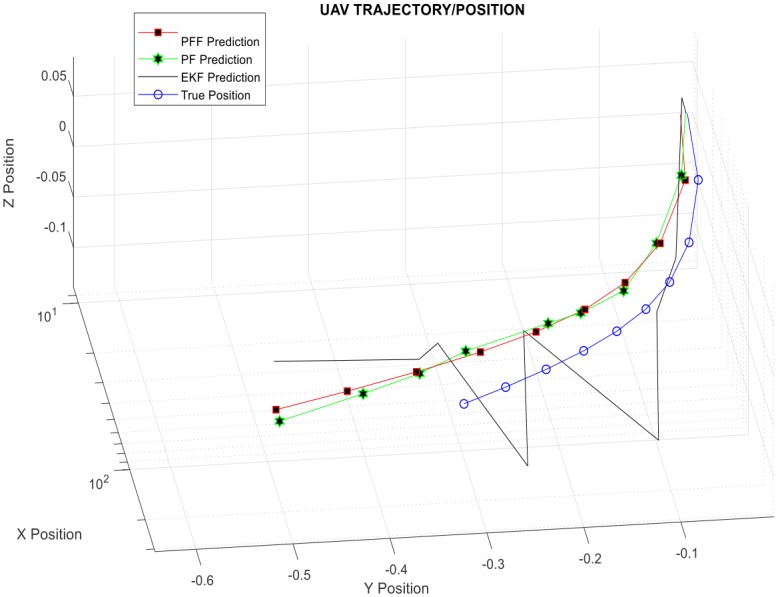
A-SLAM position predictions (zoomed version)—3D.

The trajectory (for some time interval as zoom/magnification) of an unmanned aerial system operating within the same PFF based A-SLAM scenario is depicted in Fig 7 in order to (better) see Particle Flow Filter based A-SLAM algorithm performance during research.

**Fig 8:**
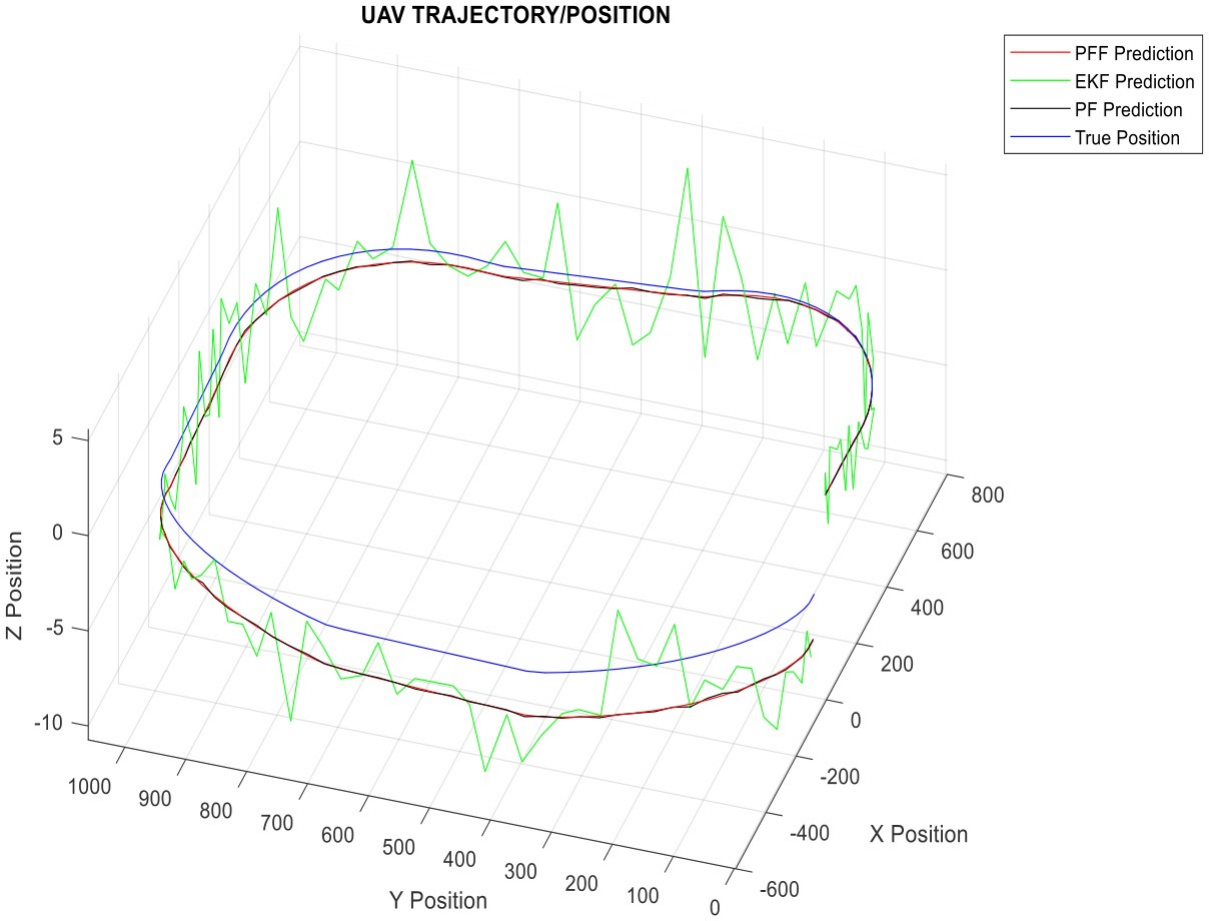
A-SLAM position predictions—3D.

Fig 8 also shows that the predictions that have the closest estimations to the actual position of the vehicle are obtained by the particle flow filter method. For a better understanding of PFF based Airborne SLAM performance, SLAM Methods Comparison in terms of Prediction Error and Process Time-Table (Table 1) is given below. It also ensures significant improvement over the particle filter in terms of calculation cost.

**Table 1:** Case study 1–performance comparison: SLAM methods prediction error and process time.

Airborne SLAM	PFF based A-SLAM	PF based A-SLAM	EKF based A-SLAM
	*Total Prediction Error*		
**X-axis (m)**	4.54	5.30	9.81
**Y-axis (m)**	5.97	6.33	10.53
**Z-axis (m)**	7.06	8.82	12.41
**Vx (m/sec)**	0.21	0.53	0.81
**Vy (m/sec)**	0.52	0.76	1.48
**Vz (m/sec)**	0.86	0.93	2.03
**Roll (rad)**	0.24	0.34	0.43
**Pitch (rad)**	0.15	0.38	0.44
**Yaw (rad)**	0.28	0.43	0.48
**Total Process Time (sec)**	173.35	185.95	166.17

The average of all error metrics, which obtained for a UAV during simulations under the same parameter conditions, also confirm the superiority of the method we proposed, as shown in Table 1. The algorithms here also compared utilizing the root-mean-square (RMS) of total error;

$$\varepsilon =\sqrt{{\sum_{x}\left(x-x_{k}\right)}^{2}}$$ (29)

**Fig 9:**
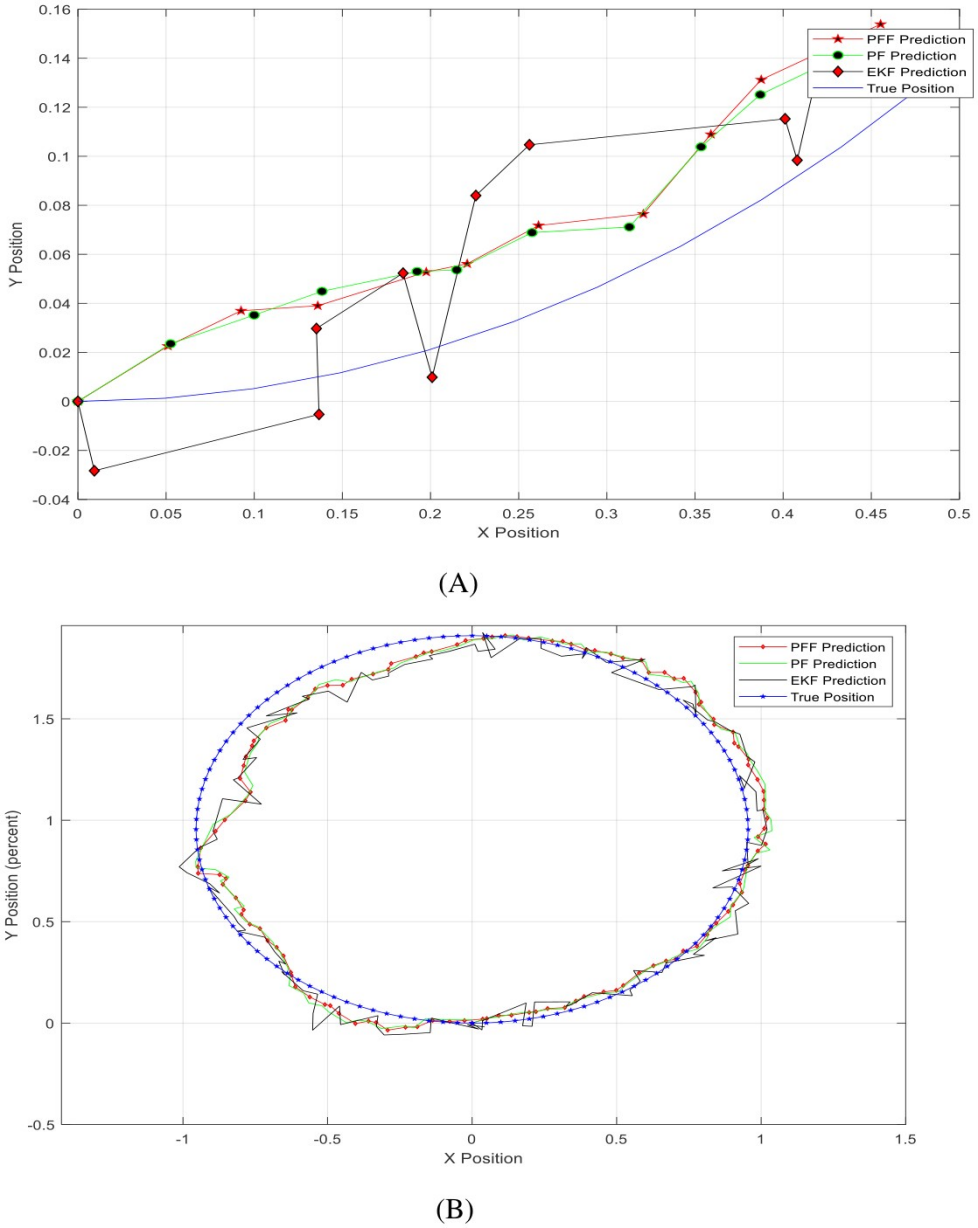
A-SLAM position predictions. (A) Short-run/zoomed version—2D. (B) Full path—2D.

Fig 9 shows A-SLAM position predictions in two-dimensional axes to see filters’ performances in different (bird-eye) view for short (Fig 9A) and long-run (Fig 9B) simulations. The figures also depicted that the predictions that have closest estimations to the actual position of the vehicle are obtained by the particle flow filter-based method proposed here for the first time.

As a footnote, it is also seen from a different point of view that the more divergent EKF filter estimates alter around the actual/real position value in the form of a control algorithm. It is also remarkable that EKF estimation performance here is worst among others. It is thought to be affected by its parameters such as noise (uncertainty) or particle number factors for others.

It is also possible to compare position estimations using Figs 7, 8 and 9, and Table 1 together. Here, as is seen, the PFF predictions are closest to real positions, while PF estimations are better than EKF ones. The system and measurement noise, particle number, migration step size, and others are among the performance parameters which will be searched in the next part.

**Fig 10:**
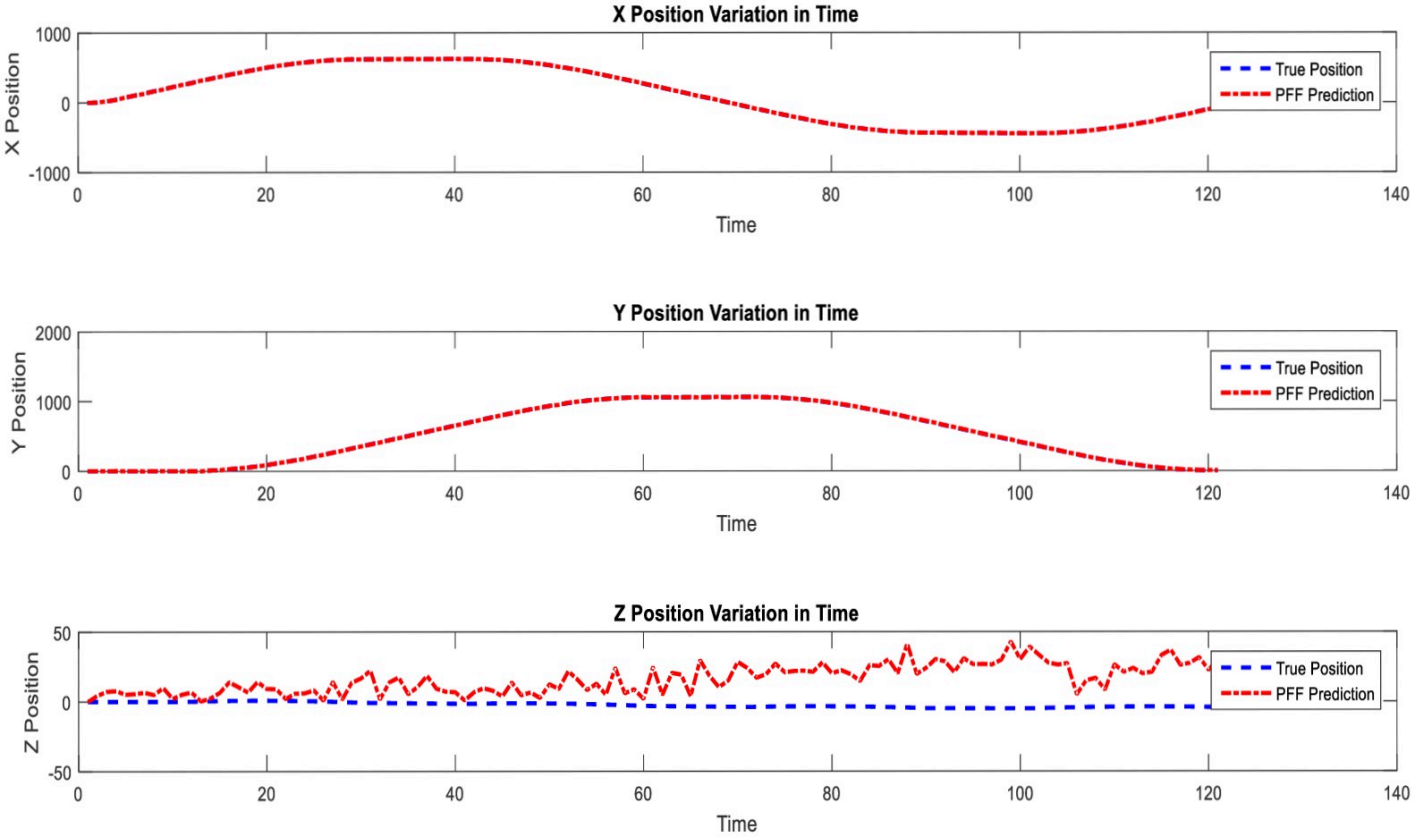
PFF-A-SLAM position predictions—1D.

**Fig 11:**
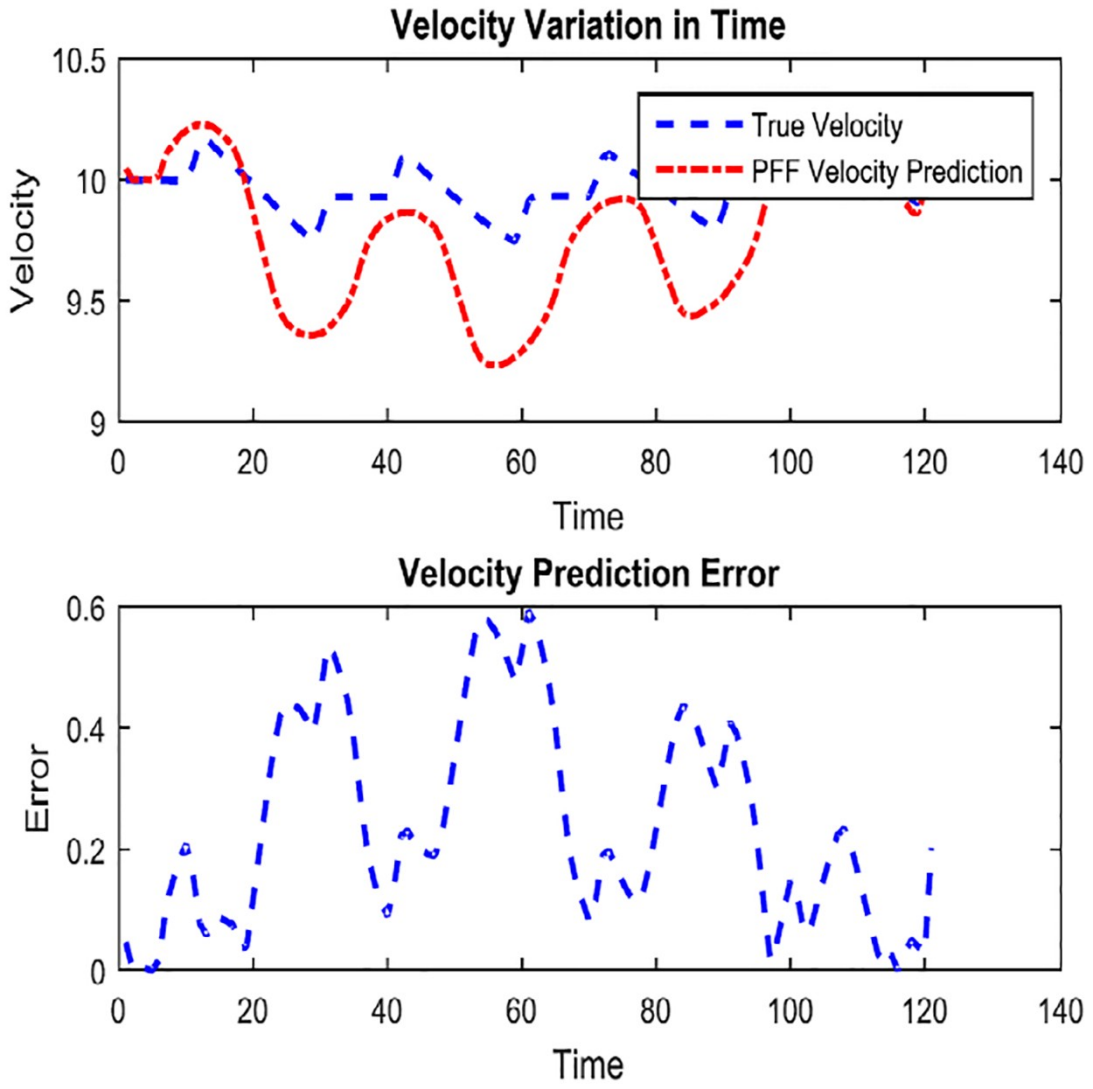
PFF based A-SLAM velocity predictions.

**Fig 12:**
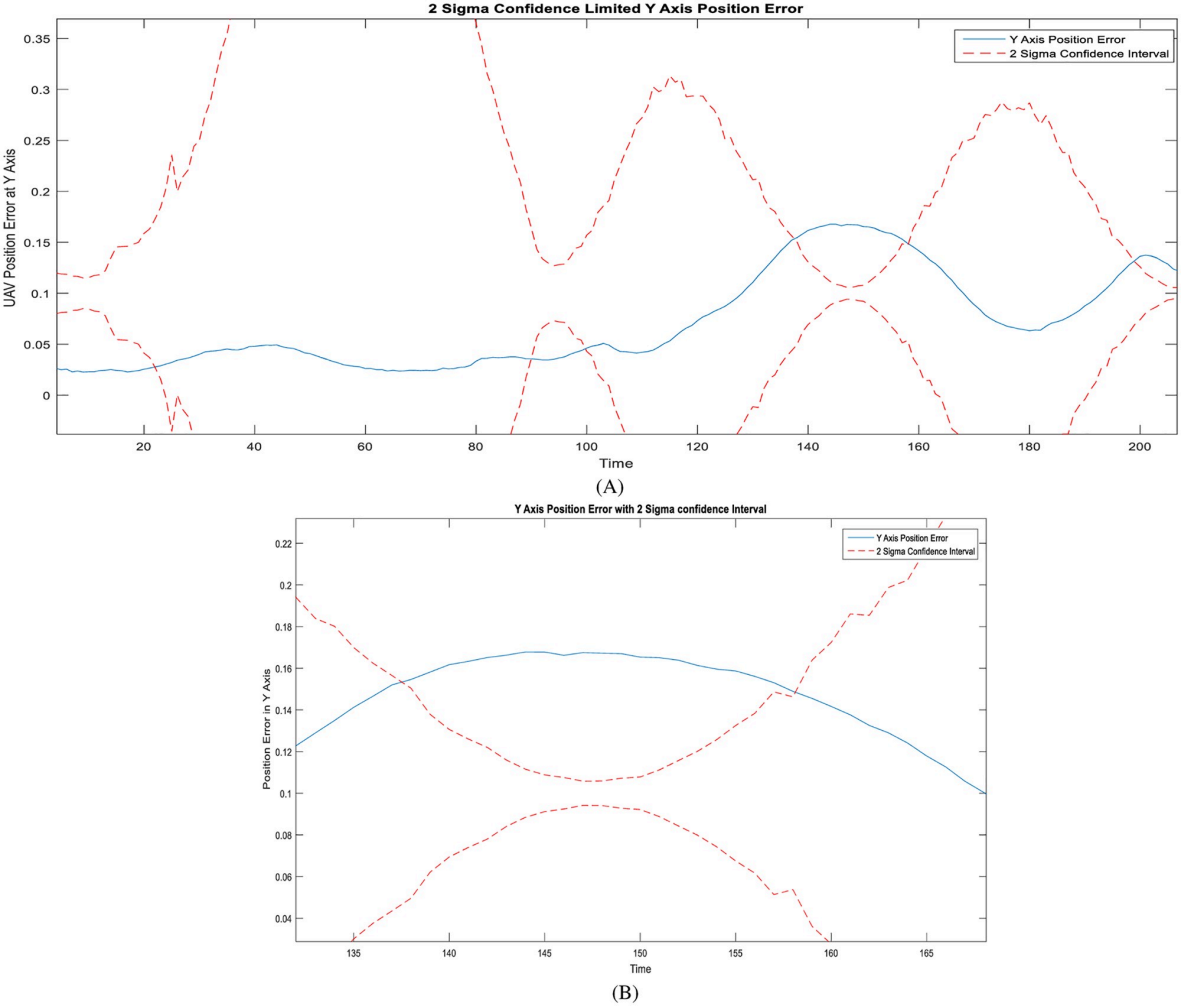
PFF-A-SLAM positions: Y-axis prediction errors with 2-sigma reliability limits. (A) Long-run. (B) The unreliable zone (zoom in).

In Figs 10, 11 and 12, performance results of simultaneous localization and mapping simulations conducted for the same curvilinear route by an autonomous aerial vehicle using particle flow filter structures are given in an example case depending on the same parameters such as noise covariances, particle count, step size, etc.

It is noteworthy to state that not for comparison but for self-evaluation of (some) performances of the Airborne Simultaneous Localization and Mapping based on the PFF simulation environment, the vehicle positions (separately) are as given in Fig 10, x axis-directional velocity changes in time as in Fig 11, and y axis-positions and reliabilities as in Fig 12. When the results above are examined, it can be concluded that the PFF-A-SLAM approach generally succeeds in position and velocity/speed estimations. However, the predictions which appear to be somewhat erratic on the z-axis may be seen as fluctuations around a constant (reference) position.

In the Simultaneous Localization and Mapping based on the Particle Flow Filter, the error arising in estimating the y-axis position of the vehicle and its zoom for some out of (2-sigma reliability limit) region is as given in Fig 12. The y variable (axis) represents the y coordinate of the vehicle position, the blue lines represent the error amount, and the red lines represent the reliability range.

Here, it is seen that the estimates of the y-axis position fall within the confidence interval. However, for some time, they are out of the confidence interval (Fig 12B), which refers to slight inaccuracy, possibly because of (again) filter unobservable states. These states (thought as landmark positions) are also planned to be investigated via observability analysis based on jacobians H and F among the next researches.

Hereby, better UAV kinematic model structure and other than fixed altitude flight simulation scenarios might have potential root-causes and solutions which are needed to be scanned in future research.

**Fig 13:**
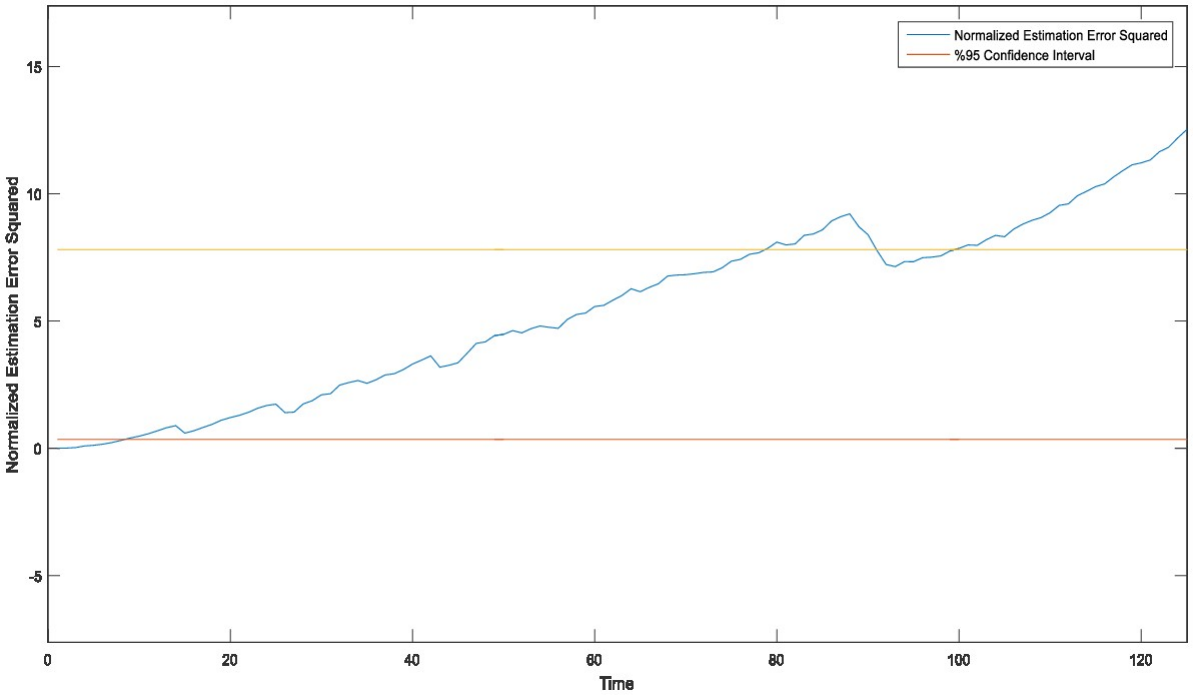
PFF based A-SLAM normalized estimation error.

Given the normal conditions set of surroundings`(without GNSS), PFF based SLAM estimation simulations for an unmanned aerial system show that the results are generally in the acceptable range for speed prediction/estimation error of UAV for a certain period (of time). However, the NEES (Normalized Estimation Error Squared for x position state) can be moved away from the 2σ confidence/reliability limits (orange/upper-red/lower lines in Fig 13), due to cumulative errors in case of long-run (simulations).

A possible reason for this could be lying in the filter inconsistency, which is considered related to parallel EKF structure and also planned to be dug out in the next research. The consistency and observability constraints may bring about unobservable subspaces, and some linearization errors of the exact version of the particle flow filter used here.

**Fig 14:**
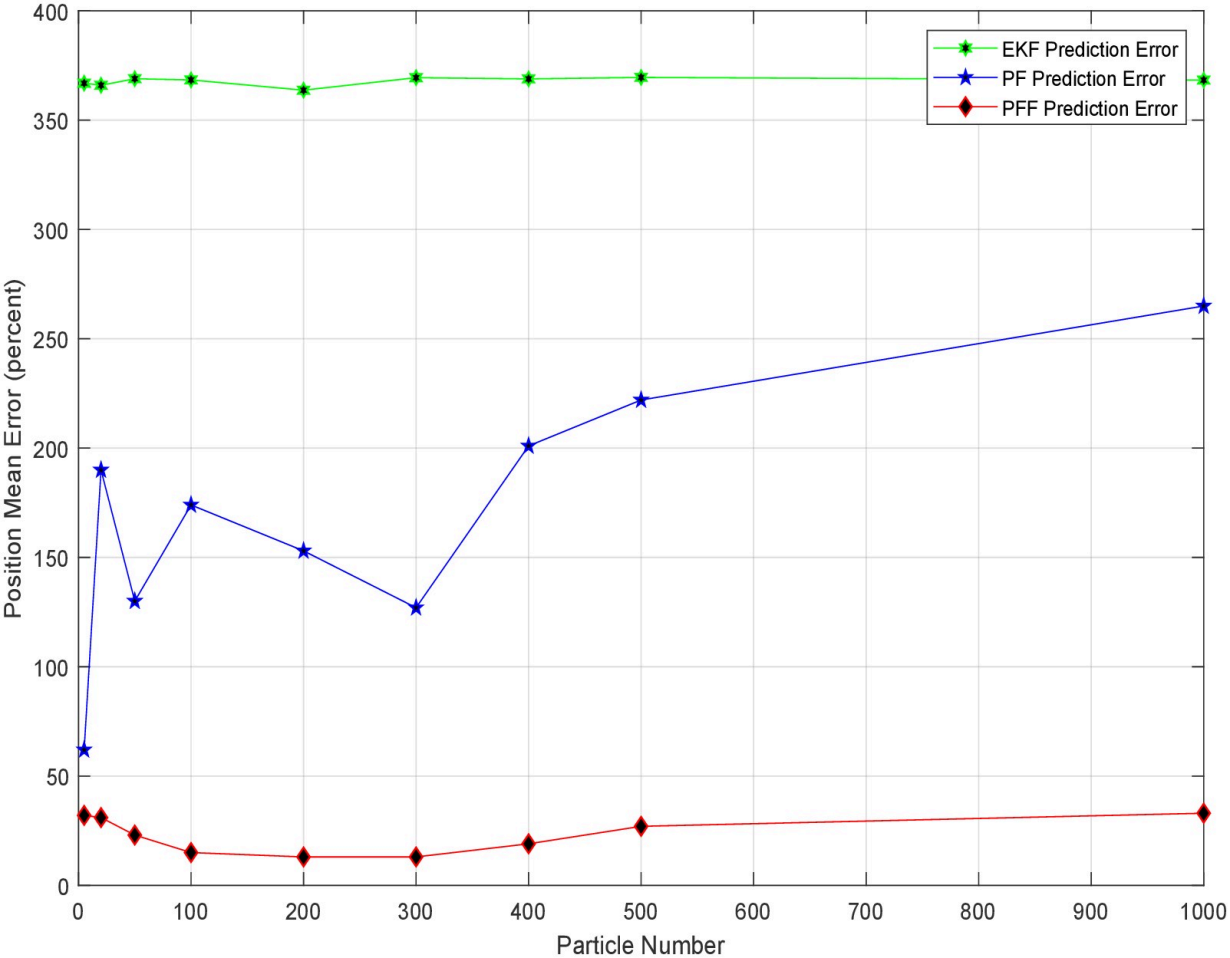
PFF-A-SLAM algorithm estimation error versus particle number.

Throughout the simulation, many different parameters investigated, and particles`number (limited to some extent) in A-SLAMs is one of them. The changes in the average number of particles versus error produced by prediction methods are shown in Fig 14. These values are obtained by taking the constant step size (1/100) for the same noise covariances above. Accordingly, the ideal number of particles is generally below 500. Here the process complexity/load for the methods is excluded from the evaluation/considerations. However, the increased number of particles will bring an additional processing burden.

**Fig 15:**
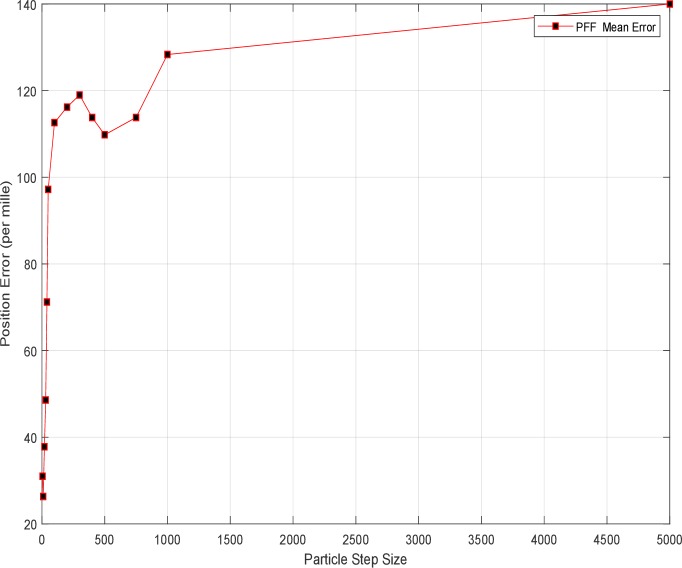
PFF-A-SLAM algorithm estimation error versus step size.

The change in migration/flow step size has also affected performance, which is determined experimentally by repetitive simulations but presented briefly. In order to determine the effect of migration amounts of particles (for posterior) to the estimation performance of the filter in the update step of the particle flow filter in the PFF-A-SLAM system is as shown in Fig 15. The practical value of the step size is again above 100 and below 500. (As for the step size, λ ranging from 0 to 1, for example, if the step size referred to 1000, it means actual step size is 1/1000 or 0.001). It is possible to say that the increase in step size is not a mitigating effect on the prediction error. However, it is sure that its rise will increase the processing burden.

Once again, at this point, it is possible to perform the methods`velocities and Eulers’ estimation error evaluation with Table 2, especially for changing noise conditions. The convergence success of the particle flow filter-based approach proposed here is seen in general if the total error is observed. It is also possible to see that more uncertain observation data, the better PFF-A-SLAM estimations, and as expected, motion uncertainty negatively affect the estimations.

**Table 2:** Case study 2–comparisons: SLAM methods’ parameters, errors and process times.

A-SLAM Algorithm	*PFF based A-SLAM*	*PFF based A-SLAM*	*PFF based A-SLAM*	*PFF based A-SLAM*	*PF based A-SLAM*	*EKF based A-SLAM*
*Number of particles*	500	500	500	500	500	N/A
*Flow step size*	1/100	1/100	1/100	1/100	1/100	N/A
*Motion uncertainty*	.001	.01	.01	.01	.01	.01
*Measurement uncertainty*	.001	.001	.01	.1	.1	.1
**Average Velocity Error**	0.72	0.76	0.82	0.86	0.93	2.03
**Average Angular Error**	0.14	0.18	0.22	0.24	0.34	0.43
**Process Time**	172.51	173.43	176.88	177.35	185.95	166.17

The load of process complexity in the PFF generally emerges in the steps of particle generation-propagations and calculations of flow parameters for each particle and the amount of migration (flow) through them. Nonetheless, the PF, in the resampling and importance sampling steps, needs calculations related to the number of particles to be processed in addition to particle generation and propagation computations. In EKF, which performs best in Gaussian noise conditions, the covariance estimation calculations based on the number of landmarks create the burden of the process (complexity and time), on a side note to its estimation convergence. The worst estimation errors of EKF for this case probably may stem from noise preference here, by the way.

## Conclusions

The idea of the operator-free platform is highly common in particularly military researches in the last few decades. Autonomous vehicles are particularly expected to perform all or part of its mission autonomously. The prerequisite of safe autonomous navigation is to detect the unmanned system location precisely; however, it is a challenge for researchers to determine the location in GNSS denied environments. Although new methods are emerging continuously, the most notable one is Simultaneous Localization and Mapping (SLAM).

The estimation methods used in SLAM problems mostly rely on parametric ones that deal with a normal distribution (defined by mean and variance) such as Kalman based filters and non-parametric state estimations (Monte Carlo or Adaptive Monte Carlo) such as particle filters. Despite Kalman and particle filter-based recursive state estimation approaches`prevalence, still, there are several attempts to finding better algorithms of estimation methods in the SLAM problem. For this reason, in this research, a novel Bayesian filtering for A-SLAM recursive state estimations called Particle Flow Filter Based Airborne Simultaneous Localization and Mapping (PFF-A-SLAM) structure is proposed to contribute for the first time in the literature. This is the initial study using particle flow filter for airborne SLAM, no other one is recorded yet. Neither drift problems in parametric methods nor particle degeneration drawbacks exist in our approach. Since we don’t impose any resampling in the PFF-A-SLAM processes, we rule out the particle degeneracy which is usually seen in A-SLAM structures using PF. This also highlights the recency of our research. It gets more successful estimations than PF, and EKF-based ones which are generally not (sufficient) enough at non-gaussian uncertainty/noise environments.

The particle flow filter, which was first proposed by Daum and Huang, is used in SLAM state estimations as a novel approach to A-SLAM here. The method includes no linearizations as in Kalman methods or no sampling/resamplings as in particle filter approaches. The application and tailoring/adaptation of particle flow filter for SLAM problem with the primer analysis here is the novelty of the study. This research also presents the mathematical background of the PFF-A-SLAM filter and model of an autonomous vehicle. The filter covariance value related to uncertainty, particle migration factor (step size), and particle numbers may be clearly stated as parameters. Those affect particle flow filter performance, which is discussed in detail according to state variables such as (UAV) positions, rates, or angles.

The simulation results presented that this novel/first-time A-SLAM applied algorithm gives very successful outcomes and has superiority over (Kalman and particle filter-based) previous methods in terms of accuracy and speed of convergence as it is clearly seen in Results and Comparisons part. Nevertheless, it has some drawbacks, notably in high-dimensional state spaces with quite low particle samples, such as computational cost/complexity (particularly for particle migrations/flows), which may cause real-time application fragilities in some circumstances. However, we think that it is solvable by PFF compatible hardware solutions such as parallel computing/processing platforms like CUDA (Compute Unified Device Architecture) or general computing approaches on GPU (Graphics Processing Unit) programming model for A-SLAM problem.

Furthermore, the Particle Flow Filter method can be preferred in the measurement environments that use low uncertainty sensors, because it gives more successful results as stated before by eliminating the problem of degeneration seen in the particle filter structure. Additionally, with these promising results, it is conceivable to use PFF for estimations in target/object-tracking, sensor fusion or radar problems, and filtering for signal/image processing in aviation, medical, or any other application areas.

For the PFF-based A-SLAM approaches, a very first step has been taken with the implementation of this (primary/initial) research. It has become a kind of motivation for further studies such as ones especially targeting real-time applications or one step before them in the high-level (GPU-CUDA/cluster) parallel programming/processing environments with high computational capacity. It is also possible to perform other ones using thread/core accelerated coding/algorithm or optimized PFF-A-SLAM structures with modified/improved particle flow approaches in future researches. However, both real-time territorial system (PFF-SLAM) and aerial system performance of (PFF-A-SLAM) the algorithm will definitely be the most important parts of further works.

In addition, the PFF-SLAM approaches for systems of water/marine environments (other than for land and air) or multi-vehicle/agent (homogenous and heterogenous) systems will also be in prospect researches. Since all these interest areas may hold other related sub-problems, it is clear that a detailed comparison of the algorithm by others will be multi-dimensional.

**Fig 16:**
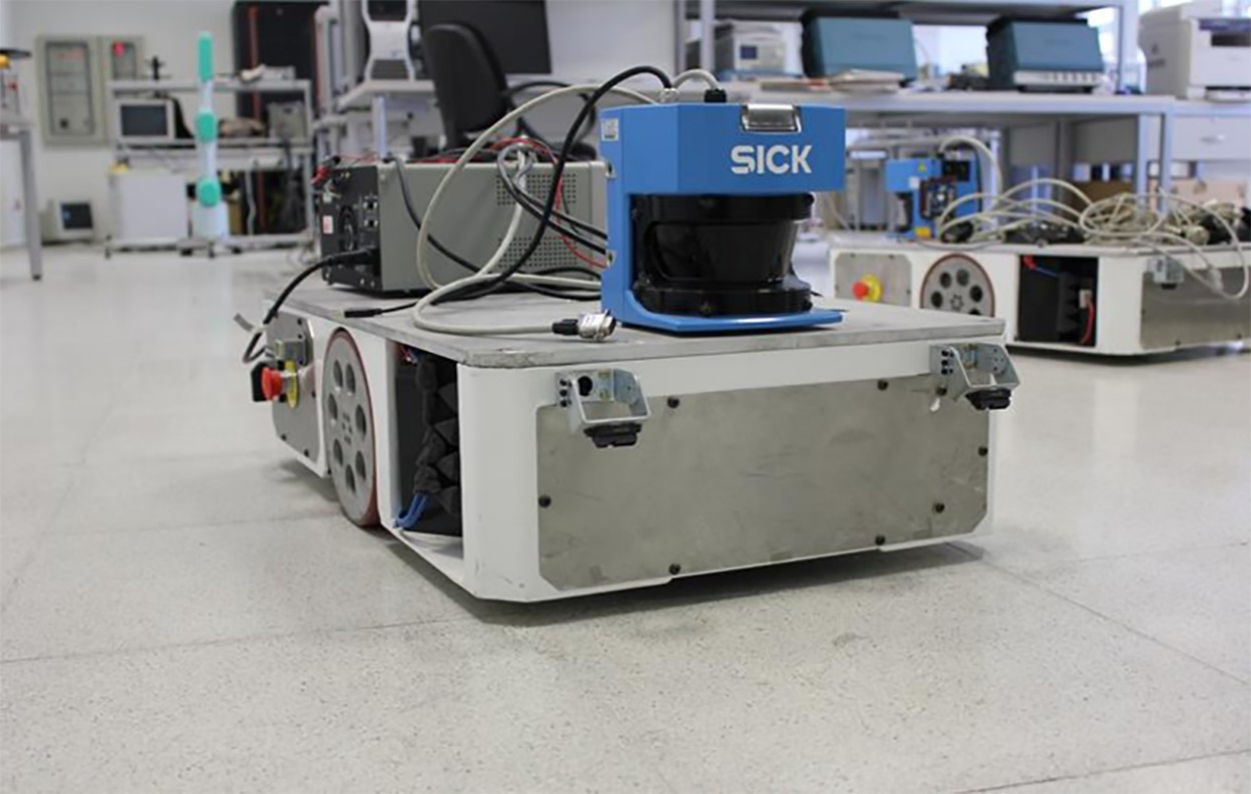
SICK-LMS511 laser range finder sensor.

**Fig 17:**
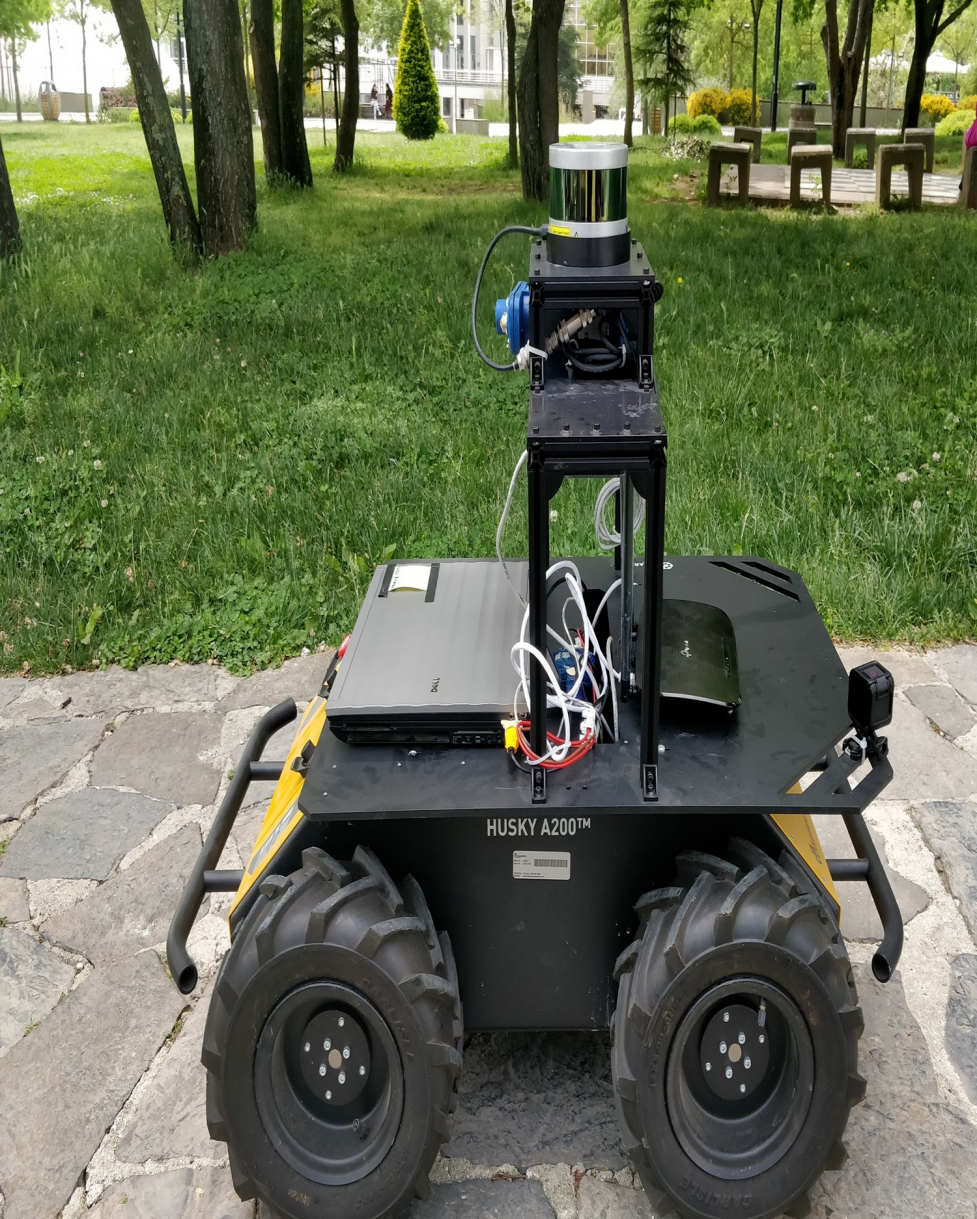
Clearpath Husky A-200 unmanned ground vehicle.

A system may include unobservable state dimensions, and it may be a waste of time to do calculations for them. Therefore, a detailed analysis of consistency and observability will be necessary for a better understanding of the algorithm's pros and cons. If the consistency and observability issues emerge as PFF-A-SLAM structure frailty by these analyses, then powered algorithms by the more consistent structures should also be needed to contribute such a promising tool in SLAM sub-problems. The estimation performance evaluation of other PFF versions (geodesic, irrotational, incompressible, etc.) by also comparing other current filters such as unscented Kalman, information filters with the help of SLAM data sets such as CSD, Zurich/Complex Urban, EuRoC, etc. in an appropriate platform scenarios using ITU Robotic Lab systems (refer to Figs 16 and 17) are also considered among the future works for better assessment of PFF in SLAM problems.
